# Electrical stimulation of the splenic nerve bundle ameliorates dextran sulfate sodium-induced colitis in mice

**DOI:** 10.1186/s12974-022-02504-z

**Published:** 2022-06-17

**Authors:** David J. Brinkman, Thomas Simon, Anne S. ten Hove, Konstantina Zafeiropoulou, Olaf Welting, Patricia H. P. van Hamersveld, Rose A. Willemze, Andrew Y. F. Li Yim, Caroline Verseijden, Theodorus B. M. Hakvoort, Misha D. Luyer, Margriet J. Vervoordeldonk, Philippe Blancou, Wouter J. de Jonge

**Affiliations:** 1 Tytgat Institute for Liver and Intestinal Research, Gastroenterology and Hepatology, Amsterdam Gastroenterology Endocrinology Metabolism, Amsterdam UMC, University of Amsterdam, Room S2-162, Meibergdreef 69, 1105 BK Amsterdam, The Netherlands; 2 Department of Surgery, Catharina Hospital, Eindhoven, The Netherlands; 3 Institut de Pharmacologie Moléculaire et Cellulaire, Université Côte d’Azur, CNRS, Nice, France; 4 Department of Pediatric Surgery, Emma Children’s Hospital, Amsterdam University Medical Centers, University of Amsterdam, Amsterdam, The Netherlands; 5 Department of Clinical Genetics, Genome Diagnostics Laboratory, Amsterdam Reproduction and Development, Amsterdam UMC, University of Amsterdam, Amsterdam, The Netherlands; 6 Galvani Bioelectronics, Stevenage, UK; 7 Department of Surgery, University of Bonn, Bonn, Germany

**Keywords:** Inflammatory bowel disease, Splenic nerve stimulation, RNA-sequencing

## Abstract

**Background:**

Vagus nerve stimulation has been suggested to affect immune responses, partly through a neuronal circuit requiring sympathetic innervation of the splenic nerve bundle and norepinephrine (NE) release. Molecular and cellular mechanisms of action remain elusive. Here, we investigated the therapeutic value of this neuromodulation in inflammatory bowel disease (IBD) by applying electrical splenic nerve bundle stimulation (SpNS) in mice with dextran sulfate sodium (DSS)-induced colitis.

**Methods:**

Cuff electrodes were implanted around the splenic nerve bundle in mice, whereupon mice received SpNS or sham stimulation. Stimulation was applied 6 times daily for 12 days during DSS-induced colitis. Colonic and splenic tissues were collected for transcriptional analyses by qPCR and RNA-sequencing (RNA-seq). In addition, murine and human splenocytes were stimulated with lipopolysaccharide (LPS) in the absence or presence of NE. Single-cell RNA-seq data from publicly available data sets were analyzed for expression of β-adrenergic receptors (β-ARs).

**Results:**

Colitic mice undergoing SpNS displayed reduced colon weight/length ratios and showed improved Disease Activity Index scores with reduced Tumor Necrosis Factor α mRNA expression in the colon compared with sham stimulated mice. Analyses of splenocytes from SpNS mice using RNA-seq demonstrated specific immune metabolism transcriptome profile changes in myeloid cells. Splenocytes showed expression of β-ARs in myeloid and T cells. Cytokine production was reduced by NE in mouse and human LPS-stimulated splenocytes.

**Conclusions:**

Together, our results demonstrate that SpNS reduces clinical features of colonic inflammation in mice with DSS-induced colitis possibly by inhibiting splenic myeloid cell activation. Our data further support exploration of the clinical use of SpNS for patients with IBD.

**Supplementary Information:**

The online version contains supplementary material available at 10.1186/s12974-022-02504-z.

## Background

Inflammatory bowel diseases (IBD) are debilitating conditions that greatly affect the daily life of patients. At present, treatment modalities for IBD mainly consist of anti-inflammatory agents with increasing intensity if disease remains uncontrolled. However, pharmaceutical treatment is accompanied with substantial side effects and has a high financial burden. In this light, novel therapies that can improve disease outcome of patients with IBD are warranted.

It is increasingly acknowledged that the autonomic nervous system possesses a regulatory activity towards the immune response. Vagus nerve stimulation (VNS) was found to improve clinical outcomes in small scale clinical trials in patients with rheumatoid arthritis and Crohn’s disease [1–3]. Similarly, in experimental colitis rat models, VNS decreased disease parameters and colonic cytokines [4–7]. Despite a positive outlook for the use VNS as immunosuppressive treatment, it has become evident that VNS affects many organs, acts on immune cells indirectly [8], and, therefore, has off-target effects, such as cardiovascular changes restricting its clinical application [9].

It has been shown that the anti-inflammatory effect of VNS in experimental endotoxemia rests on innervation of the spleen through the splenic nerve bundle [10, 11]. Accordingly, anti-inflammatory activity in experimental colitis of cholinergic agonists relies on splenic innervation [12–14]. Since the anatomical and functional connection between the vagus nerve and the sympathetic splenic nerve bundle remain topic of debate, therapeutic efficacy of nerve stimulation might be improved through directly targeting the splenic nerve bundle instead of the vagus nerve [15]. Interestingly, it has been reported that stimulation of the splenic nerve bundle could be as effective as VNS in murine models of endotoxemia and arthritis [9, 16]. Moreover, ultrasound stimulation of the splenic nerve bundle improves disease outcome in a setting of dextran sulfate sodium (DSS)-induced colitis [17]. Yet, thus far the effect of electrical splenic nerve bundle stimulation (SpNS) in experimental colitis is not well understood.

In this study, we demonstrate that electrical SpNS using implanted cuff electrodes in mice with DSS-induced colitis ameliorated colitis. Furthermore, we investigated the potential role of adrenergic receptor (AR) activation in splenic myeloid cells that could be mediating this effect.

## Methods

### Animals

Female C57BL/6 N mice (8–12 weeks) were purchased from Charles River Laboratories (Maastricht, the Netherlands). The animals were housed under specific pathogen free conditions in individually ventilated cages in the animal facility at the Amsterdam UMC, location Academic Medical Center (AMC), in Amsterdam. Animals were maintained on a 12/12 h light/dark cycle under constant condition of temperature (20 °C ± 2 °C) and humidity (55%) with ad libitum food and water. Mice handling and experimental protocols were in accordance with the local guidelines and approved by the local Animal Research Ethics Committee.

### Surgical implantation and stimulation of cuff electrodes around the splenic neurovascular bundles and the cervical vagus nerve

First, an incision was made in the skin of the head and the skull was cleaned to attach the head mount, Preci-Dip (RS components, Haarlem, the Netherlands) with dental cement (Super-Bond C&B, Sun medical, Hofmeester Dental, Rotterdam, the Netherlands) according to the manufacturer’s protocol. Second, an incision was made in the left flank to reach the spleen and to place the cuff electrode (100 µm micro cuff sling, CorTec GmbH, Freiburg, Germany) around the splenic artery, or an incision was made in the neck to expose the left cervical vagus nerve to place the cuff. The wires of the cuff were led subcutaneously to the skull and attached to the head mount. The whole procedure was performed in mice under anesthesia with 2–2.5% isoflurane/O_2_. Pre-operatively and 24 h post-operatively, meloxicam (Metacam) 1 mg/kg (Boehringer, Ingelheim am Rein, Germany) and enrofloxacin (Baytril) 10 mg/kg (Bayer Healthcare, Whippany, NJ, USA) were administered subcutaneously. Before the start of DSS treatment, mice had a recovery period of 10 days and were tethered and single housed 5 days before start of stimulation. Before start of stimulation, mice were paired based on weight and then randomly allocated (1:1) to the sham or stimulation group (Table 1). Stimulation started at the same day as the treatment with DSS and was performed 6 times per 24 h (every 4 h) for 2 min with a biphasic pulse (650 µAmp, 10 Hz, 100 µs per phase). Mice were observed during the first stimulations for behavioral changes and altered breathing patterns. Before, during and after the experiment, cuff functioning was ensured by measuring impedance using a Minirator MR Pro (NTI Audio, Essen, Germany). If the impedance was > 25 kΩ before start of stimulation, the mouse was allocated to the sham group. Animals that received sham stimulation underwent the same surgical procedure with cuff placement, were tethered to a wire but no actual stimulation was performed.

**Table 1:** Impedance measurements

Mouse ID	Impedance according to manufacturer	Impedance before DSS	Impedance during DSS	Impedance after DSS	Group
#26	3.7	10	11	11	Stim
#27	4.6	13			Sham
#28	4.8	13			Sham
#29	3.6	18	14	15	Stim
#30	4.5	18			Sham
#31	6.8	17	14	13	Stim
#32	4.8	> 50			Sham
#33	5.1	16			Sham
#34	3.6	9.7	11	11	Stim
#35	4.4	14			Sham
#36	3.9	18	14	14	Stim
#37	4.6	21			Sham
#38	3.6	15	14	13	Stim
#39	5.1	26			Sham
#40	3.9	19	13	12	Stim
#41	5.1	18	14	14	Stim
#42	5.9	20	21	1	Stim
#43	3.9	16	16	15	Stim
#44	4.6	20			Sham
#45	4.3	2.4			Sham
#46	6.9	17	15	14	Stim
#47	4.1	20			Sham
#48	4.6	17			Sham
#49	4.2	18	14	13	Stim
#50	4.6	17	16	16	Stim
#51	4.8	27			Sham
#52	4.8	18	15	16	Stim
#53	6.2	14	11	12	Stim
#54	5.1	35			Sham
#55	5.1	> 50			Sham
#56	4.8	23			Sham

*DSS* dextran sulfate sodium

### Surgical splenic denervation

Via a midline incision the spleen and splenic artery were located. Selective denervation was achieved by cutting the catecholaminergic nerve fibres running along the splenic artery. Sham-operated animals underwent a laparotomy without denervation. Surgery was performed on anesthetized mice by injecting intraperitoneally (i.p.) a mixture of fentanylcitrate/fluanisone (Janssen, Beerse, Belgium) and midazolam (Roche, Woerden, the Netherlands). Finadyne (Intervet, De Bilt, the Netherlands) was injected subcutaneously pre- and postoperatively. Before the start of the DSS-induced colitis experiment, mice had a recovery period of 2 weeks.

### Acute SpNS and endotoxemic shock

Seven days following implantation, animals were injected i.p. with a lethal dose of lipopolysaccharide (LPS; 400 µg). Electrostimulation was applied using a PlexStim V2.3 (Plexon) starting at − 10, 0 and + 20 min relative to LPS injection. Mice were electrically stimulated with the same parameters as above at 16, 20, 24, 30, 34 and 38 h after LPS injection. Survival was followed over 4 days. Serum was collected at 90 min after LPS injection and assessed for Tumor Necrosis Factor (TNF)-α levels. Again, controls were fully Cortec implanted mice, which did not receive electrical stimulation (sham). Electrostimulation were rectangular charged-balanced biphasic pulses (650 μAmp, 10 Hz, 100 μs per phase) for 2 min. For TNF-α, retro-orbital blood sampling was performed under isoflurane anesthesia. TNF-α levels were measured by ELISA (Mouse TNF-alpha DuoSet, R&D Systems) following manufacturer instructions.

### Dextran sulfate sodium (DSS)-induced colitis

Two percent (w/v) DSS (TdB Consultancy, Uppsala, Sweden) was added to the drinking water for 5 consecutive days. Daily replacement of drinking water with fresh DSS solutions was performed. After 5 days, DSS drinking water was replaced by normal drinking water and animals were followed for weight and behavior for the subsequent 7 days, making the total experiment length 12 days. During the study, bodyweight was monitored daily. At the end of the study, mice were sacrificed, and the colon weight and length were measured, as parameter for colitis. Then, the tissue was snap-frozen in liquid N_2_ and stored at − 80 °C or put in 10% formalin for further processing. The disease activity index (DAI), ranging from 0 to 9, was used to assess the clinical outcome of the DSS-induced colitis. DAI was determined by combining scores of stool consistency (0–3), occult blood in the stool (0–3), and macroscopic inflammation (0–3) [18].

Endoscopy was performed at day 8 after start of DSS. Endoscopy was performed under anesthesia with 3% isoflurane/O_2_ to assess colonic inflammation. The Olympus URF type V endoscope (Zoeterwoude, the Netherlands) was rectally inserted for a maximum of 5 cm and videos of the endoscopy were recorded using a Medicap USB200 Medical Digital Video Recorder (Roermond, the Netherlands), while retracting the endoscope. A blinded and trained technician determined the murine endoscopic index of colitis severity (MEICS), consisting of wall thickening, vascularity, visible fibrin, granularity, and stool consistency, with each component scoring between 0 and 3 [19].

### Histology

Swiss rolls of the distal colon were fixated in 10% formalin. Afterwards the tissue was embedded at the Pathology department of the Amsterdam UMC, location AMC, in paraffin for routine histology. A blinded and experienced pathologist evaluated formalin-fixed hematoxylin and eosin (HE) stained tissue sections microscopically. The pathologist scored the distal colon based on eight characteristics of inflammation [20]. This resulted in a total histology score ranging from 0 to 24.

### RNA isolation and RNA sequencing analysis

RNA was extracted from snap-frozen colon tissue after homogenization of the samples in TriPure isolation reagent (Roche Applied Science, Almere, the Netherlands) according to the manufacturer’s instructions. For RNA sequencing (RNA-seq), RNA quality was assured using the Bioanalyzer (Agilent, Santa Clara, USA), where samples with an RIN score of > 8 were used for further analyses. mRNA was converted into cDNA with the KAPA mRNA HyperPrep Kit (Roche), whereupon the cDNA was prepared for sequencing on the HiSeq4000 at the Core Facility Genomics, Amsterdam UMC, in a 50 bp single-ended fashion to a depth of 40 million reads per sample. The raw reads were checked for quality using FastQC (v0.11.8) and MultiQC (v1.7) [21, 22] and were subsequently mapped using STAR (v2.7.3) against mouse genome GRCm38 [23]. Post-alignment processing was done using SAMtools (v1.9),[24], whereupon reads were counted using featureCounts as found in the Subread package (v1.6.4) [25, 26]. Gene features were obtained from Ensembl (v98) [27]. Resulting counts were imported and analyzed in the R statistical environment (v3.6.2) using packages obtained from Bioconductor (v3.10) [28]. DESeq2 was used to perform the pairwise differential expression analyses [29]. Plots were made using pheatmap (v1.0.12) and ggplot2 (v3.3.1) [30, 31].

### cDNA synthesis and quantitative PCR analysis

For quantitative polymerase chain reaction (qPCR), RNA was further cleaned from DSS with the Bioline ISOLATE II RNA mini kit (GC biotech B.V., Alphen a/d Rijn, the Netherlands). cDNA was synthesized using dNTPs (ThermoFisher Scientific, Landsmeer, the Netherlands), Random primers (Promega, Leiden, the Netherlands), Oligo dT primers (Sigma, Zwijndrecht, the Netherlands), Revertaid and Ribolock (ThermoFisher Scientific) according to the manufacturer’s instructions. PCR was performed using SensiFAST SYBR No-ROX (GC biotech B.V.) on a LightCycler 480 II (Roche Applied Science) to analyze expression levels of genes of interest using LinRegPCR software [32]. For normalization the reference genes hypoxanthine phosphoribosyltransferase (*H*prt), cyclophilin, Non-POU Domain Containing Octamer Binding (*Nono*) and ribosomal protein lateral stalk subunit P0 (*Rplp0*) were selected, after analysis for stability in geNorm [33]. Primers (synthesized by Sigma) are listed in Table 2.

**Table 2:** Primer sequences for qPCR

Gene	Forward sequence	Reverse sequence
IL-1β	GCCCATCCTCTGTGACTCAT	AGGCCACAGGTATTTTGTCG
Il-6	GAGTTGTGCAATGGCAATTCTG	TGGTAGCATCCATCATTTCTTTGT
Il-10	TGTCAAATTCATTCATGGCCT	ATCGATTTCTCCCCTGTGAA
Il-12	AGACCCTGCCCATTGAACTG	CGGGTCTGGTTTGATGATGTC
TNF-α	TGGAACTGGCAGAAGAGGCACT	CCATAGAACTGATGAGAGGGAGGC
Mmp-7	TCTGCATTTCCTTGAGGTTG	AGGAAGCTGGAGATGTGAGC
Acod-1	ACTCCTGAGCCAGTTACCCT	GGTGGTTCACTTTCAAGCCG
Cxcl1	CCACACTCAAGAATGGTCGC	TCTCCGTTACTTGGGGACAC
Cxcl2	CCCAGACAGAAGTCATAGCCAC	TGGTTCTTCCGTTGAGGGAC
S100a8	ACTTCGAGGAGTTCCTTGCG	TGCTACTCCTTGTGGCTGTC
S100a9	TGGGCTTACACTGCTCTTACC	GGTTATGCTGCGCTCCATCT
Hprt	CCTAAGATGAGCGCAAGTTGAA	CCACAGGACTAGAACACCTGCTAA
Cyclophilin	ATGGTCAACCCCACCGTGT	TTCTGCTGTCTTTGGAACTTTGTC
Nono	AAAGCAGGCGAAGTTTTCATTC	ATTTCCGCTAGGGTTCGTGTT
Rplp0	CCAGCGAGGCCACACTGCTG	ACACTGGCCACGTTGCGGAC

### Protein concentrations of cytokines in intestinal tissue

Snap-frozen colon tissue was homogenized on ice in Greenberger Lysis Buffer (150 mM NaCl, 15 mM Tris, 1 mM MgCl·6H_2_O, 1 mM CaCl_2_, 1% Triton) with protease inhibitor cocktail (Roche Applied Science), pH 7.4, diluted 1:1 with phosphate buffered saline (PBS). In these tissue lysates, protein concentrations of IL-6, IL-10, IL-12, TNF-α, interferon (IFN)-γ and monocyte chemoattractant protein (MCP)-1 were measured with a mouse inflammation kit by BD cytometric bead assay (CBA; BD Bioscience, San Jose, CA, USA) according to manufacturer’s protocol, with the exception that reagents were 10 times diluted.

### Adrenergic cell culture assays

Murine spleens were obtained from female C57BL/6 mice. Human splenic tissue was obtained from patients that underwent distal pancreatectomy for pancreatic cancer during which the spleen was taken out as part of the procedure. Splenic tissue was obtained in consultation with a pathologist to assure that tissue was not affected by any tumorous tissue. The Medical Ethics research Committees United (MEC-U, Nieuwegein, the Netherlands) approved the protocol (registration number W16.182) and patients provided informed consent for the use of their material. Spleens or splenic tissue were immediately homogenized on a 70 µm cell strainer and suspended in RPMI-1640 medium (supplemented with 10% fetal calf serum (FCS; Bodinco, Alkmaar, the Netherlands), 100 U/ml pen/strep (Lonza, Basel, Switzerland), 2 mM L-Glutamine (ThermoFisher Scientific) to obtain a single cell suspension. Cells were counted using the Coulter Counter (Beckman Coulter, Indianapolis, USA) and were plated using 1*10^6^ cells per well. All conditions were performed in triplicate. Cells were pre-treated with norepinephrine (NE) and/or propranolol (both 1 mM, Sigma-Aldrich, Saint Louis, USA) 30 min before exposure to LPS 100 ng/mL (Bio-Connect, Huissen, the Netherlands). Murine TNF-α was assessed after 4 h in supernatant with ELISA (R&D systems, Minneapolis, USA) according to the manufacturer’s instructions. Human cytokines (TNF-α, IL-6, and IL-8) were assessed by means of BD cytometric bead assay (CBA; BD Bioscience, San Jose, CA, USA) according to manufacturer's protocol, with the exception that reagents were 10 times diluted.

### Single-cell RNA-sequencing database studies

Publicly available single-cell RNA-sequencing (scRNA-seq) data sets from two C57BL/6JN mouse spleens were downloaded from the Gene Expression Omnibus (GEO; GSE109774) [34]. Alignment was done against GRCh38 using Cell Ranger Software (v3.1.0; 10 × Genomics, Inc., Pleasanton, CA, USA) and subsequently data sets were imported in Rstudio (v1.3.1093) [35]. Seurat was used to import, integrate, and cluster data, and plots were made with ggplot2 (v3.3.3).[31, 36] Cells were filtered for dead cells that were identified by a low (500–5000) gene count. Clusters were identified by Louvain clustering method, and annotation was performed with use of known markers [34]. Thereafter, expression of *Adrb2* (encoding for β2-AR) was assessed. The top 13 principal components were used to calculate the t-distributed stochastic neighbor embedding (t-SNE).

### Data presentation and statistical analysis

Graphs and statistical analyses were made with Prism 8.3 (GraphPad Software, La Jolla, CA, USA). For all data, a Kolmogorov–Smirnov test was used to determine normality of distribution. Data are shown as mean (if distributed normally) plus standard deviation or median (if not distributed normally), and individual data points. A *P* value < 0.05 was considered significant. Data were compared with the independent *t* test or Mann–Whitney *U* test as appropriate. Survival was plotted using Kaplan–Meier’s curves and differences between groups were estimated using the log-rank test. In case of multiple groups, a one-way ANOVA and Dunnett’s multiple comparison test were used.

## Results

**Fig. 1:**
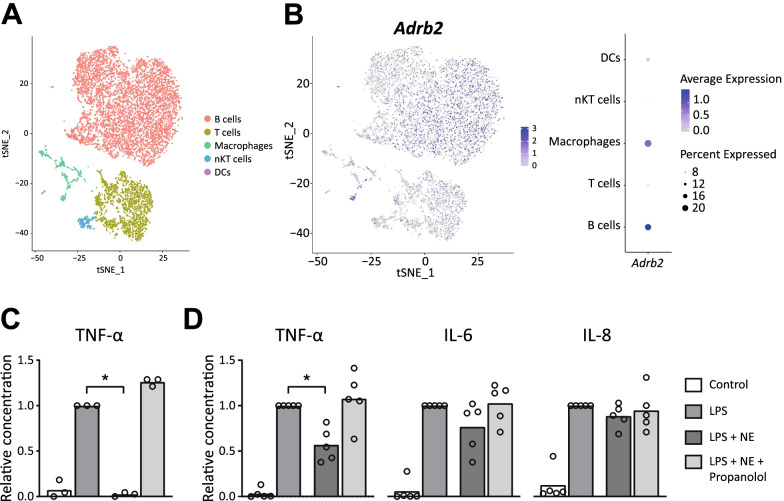
Distribution of *Adrb2* in murine splenocytes and decrease of pro-inflammatory cytokines by NE in murine and human splenocyte cultures. **A** Cell type clusters visualized by t-SNE clustering (color coding) based on the expression of known marker genes. **B** t-SNE and dotplot showing *Adrb2* expression per cell type. *N* = 2 mice. **C** Protein levels of TNF-α in supernatant from murine splenocytes after treatment with LPS and pretreatment with norepinephrine and/or propranolol. *N* = 3 mice. **D** Protein levels of TNF-α, IL-6 and IL-8 in supernatant from human splenocytes after treatment with LPS and pretreatment with norepinephrine and/or propranolol. *N* = 5 subjects. For both murine and human data, protein levels were relative compared with LPS condition. * indicates a significant difference compared to LPS treated splenocytes. Statistical differences were assessed with a one-way ANOVA and Dunnett’s multiple comparison test. *t-SNE* t-distributed stochastic neighbor embedding, *nKT* natural killer T cell, *DC* dendritic cell, *Adrb2* adrenergic receptor β2, *TNF* tumor necrosis factor, *IL* interleukin, *LPS* lipopolysaccharide, *NE* norepinephrine

Recently, it was shown that SpNS could decrease disease severity in a mouse model of arthritis and that SpNS causes the release of splenic NE, which in turn can reduce monocyte and macrophage LPS-induced cytokine secretion via β2-AR [9]. A much debated issue is whether NE targets β2-AR on macrophages or T cells that produce acetylcholine [8]. Therefore, the expression of *Adrb2* in murine splenocytes was examined using a publicly available scRNA-seq data sets. A total of 13,365 features were retrieved in 9568 individual cells. After quality control, the remaining 9407 cells were annotated to main cell populations (i.e., T, B, natural killer cells (NK), dendritic cells, and macrophages) using previously reported markers (Fig. 1A) [34]. Subsequent feature analysis showed a marked expression of *Adrb2* in a cluster of cells belonging to the B cell population, when compared with other cell populations (log2FoldChange = 0.51, padj = 8.98E-18). In addition, *Adrb2* expression by a cluster of cells belonging to the macrophage population was found to be significantly increased when compared to other cell populations (log2FoldChange = 0.31, padj = 0.009) (Fig. 1B).

Hereafter, LPS-induced inflammatory activation on both murine and human splenocyte cultures was investigated. In line with earlier investigations, NE decreased the release of TNF-α following LPS challenge in murine and human splenocytes. This effect was abrogated by pre-treatment with propranolol, demonstrating a mechanism involving β-ARs (Fig. 1C, D).

**Fig. 2:**
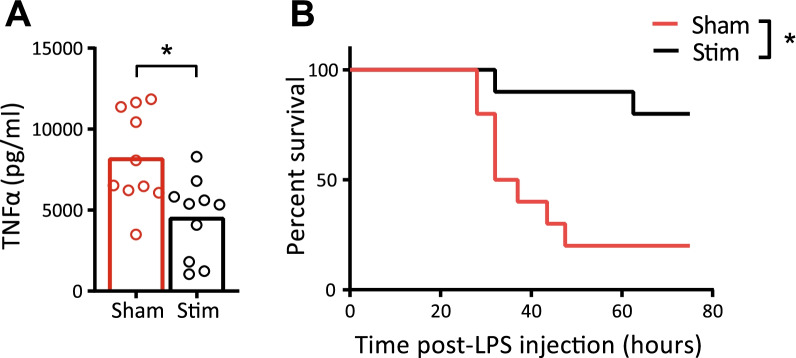
SpNS reduces systemic TNF-α and survival after LPS injection. **A** Systemic TNF-α levels 90 min after LPS injection. **B** Survival after LPS injection. *N* = 4–10 per group. Data are expressed as mean and individual data points. *indicates a significant difference compared to control. Statistical differences between Sham and Stim mice were assessed using an independent *t*-test. Statistical difference in survival was assessed using the log-rank test. *P* < 0.05 was considered significant. *SpNS* splenic nerve plexus stimulation, *TNF* tumor necrosis factor; *LPS* lipopolysaccharide

**Fig. 3:**
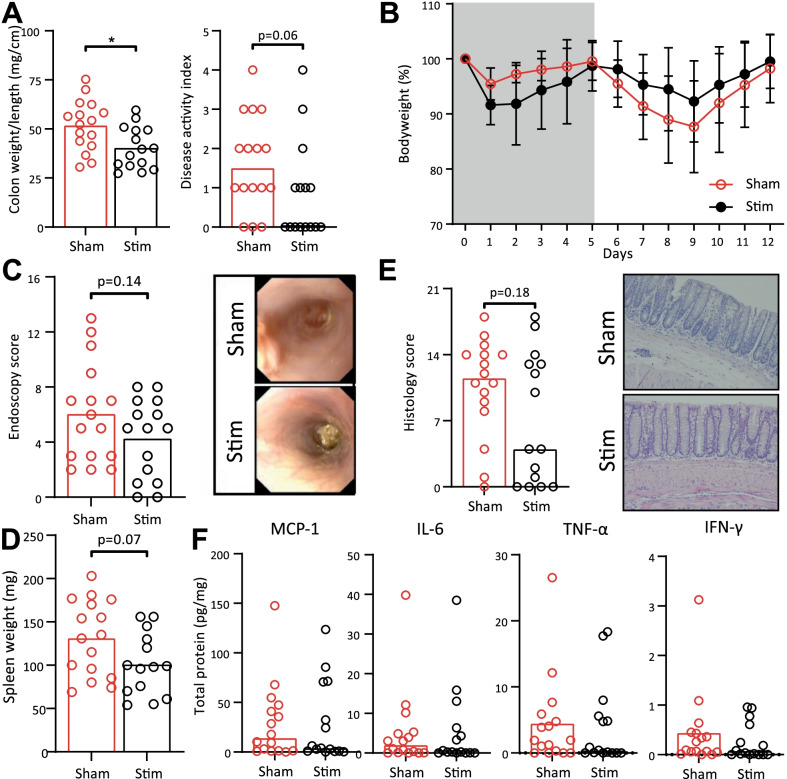
SpNS improves DSS-induced colitis. **A** Colon weight/length and Disease Activity Index at day 12. **B** Bodyweight loss of mice over time, compared with day 0. Data are expressed as mean and SD. **C** Endoscopy score at day 8 and two representative images. **D** Spleen weight. **E** Histology score and two representative images are shown of a hematoxylin and eosin staining, magnification 10×. **F** Protein levels of MCP-1 IL-6, TNF-α and IFN-γ in colon homogenates, normalized for total protein levels. *N* = 15–16 per group. All mice (both sham and stim) were implanted with a cuff electrode, were allowed to recover for 10 days and then received DSS in drinking water for 5 days followed by a 7 day recovery period. Data are expressed as mean or median and individual data points. Statistical differences between sham and stim mice were assessed using an independent t-test or a Mann–Whitney *U* test. *P* < 0.05 was considered significant. *DSS* dextran sulfate sodium, *SpNS* splenic nerve plexus stimulation, *MCP* monocyte chemoattractant protein, *IL* interleukin, *TNF* tumor necrosis factor, *IFN* interferon

As earlier studies demonstrated that vagotomy aggravates colitis outcome in a DSS-induced animal model through splenic innervation, and VNS ameliorates Crohn’s disease in patient trials, we hypothesized that disrupting the splenic nerve bundle (SplX) would worsen colitis [37]. As expected, mice receiving DSS in drinking water lost weight, showed an increased DAI and had a shortened colon due to edema compared to control mice (Additional file 1). The expression of inflammatory colonic cytokines was also increased. However, splenic nerve bundle denervation did not alter these outcomes significantly when compared to sham-operated animals. Hence, we could not demonstrate a tonic and intrinsic role for splenic innervation for regulation of immune responses in this model. Notably, VNS using chronic nerve cuffs around the left cervical vagal branch did not ameliorate disease in the setting of DSS-induced colitis (Additional file 2). To address the role of splenic nerve activity in the immune response in DSS-induced colitis, we next assessed the effect of SpNS by implanting a cuff around the splenic artery and surrounding nerve bundle (Additional file 3). Electrical stimulation of the nerve bundle can be varied in amplitude, current, and frequency. Using the stimulation parameters depicted in Additional file 3, SpNS led to a reduced induction of systemic TNF-α and increased survival in LPS-injected mice (Fig. 2A, B). Using the stimulation parameters established, we next implanted cuff electrodes around the splenic nerve bundle and artery and tested the stimulation regiment in conscious, freely moving mice, in the setting of DSS-induced colitis. SpNS applied 6 times daily for 12 days reduced the colon weight/length ratio (40.4 vs. 51.8 mg/cm; *P* = 0.01) and DAI (0 vs. 1.5; *P* = 0.06) when compared with sham stimulated mice (Fig. 3A). No clear difference in the loss of bodyweight over time was observed (Fig. 3B). At day 8, during the peak of DSS-induced colitis, comparable endoscopic scores (4.3 vs. 6.1; *P* = 0.14) were found in the animals treated with SpNS vs. animals that underwent sham-stimulation (Fig. 3C). At the end of the experiment, spleen weight (98.5 vs. 133.0; *P* = 0.07), histology scores (4 vs. 11.5; *P* = 0.18), and protein expression of cytokines in the colon were not significantly different between mice that received SpNS and sham stimulated mice, although trends towards amelioration of colitis in SpNS-treated mice were evident (Fig. 3D–F).

**Fig. 4:**
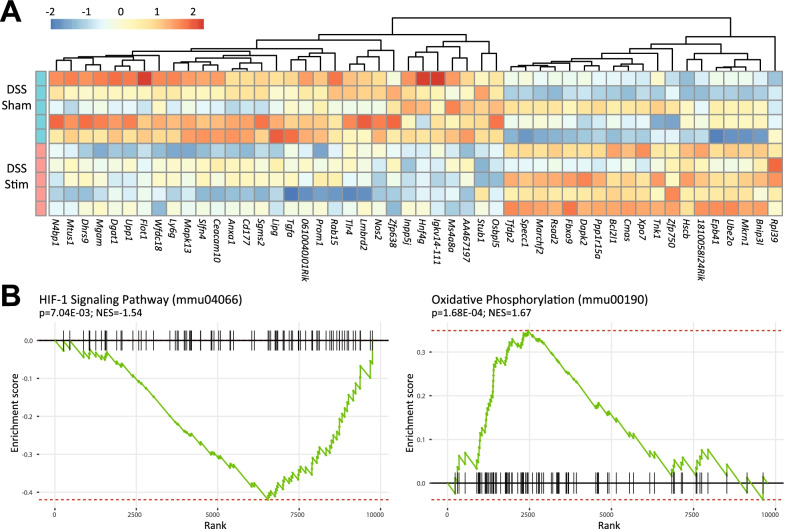
SpNS induces transcriptomic changes in the spleen. **A** Heatmap of the top 50 differentially expressed genes when comparing sham and SpNS mice. **B** GSEA plots showing depletion and enrichment of HIF-1 signaling pathway and Oxidative phosphorylation pathway associated genes among the SpNS mice. *SpNS* splenic nerve plexus stimulation, *GSEA* gene set enrichment analysis, *NES* normalized enrichment score, *HIF* hypoxia-inducible factor

Because SpNS ameliorated clinical features of colitis, we aimed to identify molecular events following SpNS. Transcriptional profiling by means of RNA-seq was performed on spleens from mice with DSS-induced colitis that received sham stimulation and mice with DSS-induced colitis that received SpNS. The top 50 genes that were differentially expressed between sham stimulated mice and SpNS mice are listed in Fig. 4A. The downregulation of nitric oxide synthase 2 (*Nos2*) and gene signatures associated with immune metabolism prompted us to further focus on genes and pathways that are involved in cellular immune metabolism, which is known to be influenced by β2-AR activation [38]. For instance, Hypoxia-Inducible Factor (HIF)-1α marks the cellular response to systemic oxygen levels in various cells, including activated macrophages [39]. Expression of genes in the HIF-1α signaling pathway was significantly reduced in SpNS spleens (*P* = 0.007), while expression of genes encoding proteins relevant to oxidative phosphorylation was relatively increased in SpNS spleen cells (*P* < 0.001, Fig. 4B). This suggests that SNS favors the metabolic state of oxidative phosphorylation over glycolytic states in stimulated splenocytes, corresponding to our earlier observation of NE-simulated macrophages [38] and characteristics of macrophages found in tissue mucosa of colitis in remission [40].

Next, to explore the underlying mechanisms through which SpNS affected intestinal inflammation, we performed bulk RNA-seq. Differentially expressed genes in colon were assessed between mice that did not receive stimulation or DSS, sham stimulated mice with DSS-induced colitis, and SpNS-treated mice with DSS-induced colitis (Additional file 4). Comparing the transcriptomes of mice with DSS-induced colitis (without SpNS) and control mice indicated an expected induction of genes encoding pro-inflammatory cytokines, such as TNF-α, and genes involved in wound healing and remodeling, such as Matrix Metalloproteinase (MMP)-7 (Additional file 5).

**Fig. 5:**
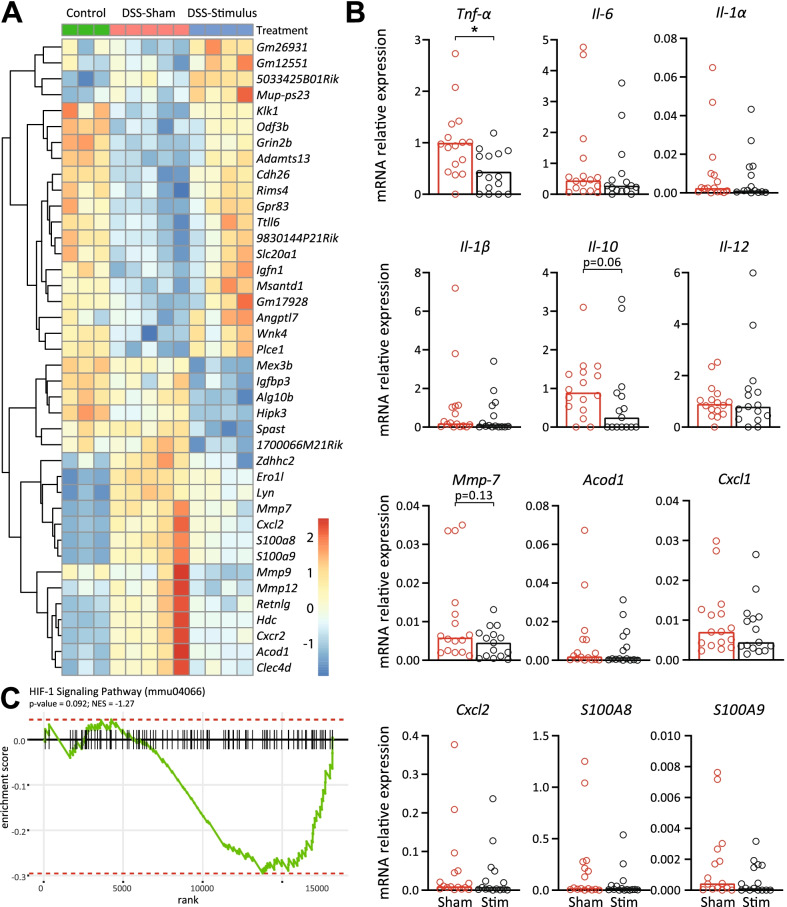
SpNS induces transcriptomic changes in the colon. **A** Heat map of top 40 differently expressed genes when comparing between sham and SpNS mice. **B** mRNA levels of TNF-α, IL-6, IL-1α, IL-1β, IL-10, IL-12, MMP-7, ACOD1, CXCL1, CXCL2, S100A8 and S100A9. mRNA levels are normalized against reference genes *Nono* and *RPLP0*. *N* = 15–16 per group. **C** GSEA plots showing depletion of HIF-1 signaling pathway associated genes among the SpNS mice. Data are expressed as mean (TNF-α) or median (other) and individual data points. Statistical differences between sham and stim mice were assessed using an independent *t*-test or a Mann–Whitney *U* test. *P* < 0.05 was considered significant. *DSS* dextran sulfate sodium, *SpNS* splenic nerve plexus stimulation, *HIF* hypoxia-inducible factor, *Nono* non-POU domain-containing octamer-binding protein, *RPLP0* ribosomal protein lateral stalk subunit P0, *TNF* tumor necrosis factor, *IL* interleukin, *MMP* matrix metalloproteinase, *ACOD* cis-aconitate decarboxylase, *CXCL* chemokine (C-X-C motif) ligand, *S100* S100 calcium-binding protein, *GSEA* gene set enrichment analysis

When comparing sham stimulated and SpNS mice with DSS colitis, 87 genes were differentially expressed in the colonic tissue of which the top 40 are shown in Fig. 5A. Genes that were significantly different were predominantly found in the domains of leukocyte chemotaxis and migration. Noteworthy is the SpNS-induced downregulation of S100A8 and S100A9, which encode subunits of calprotectin, the clinical biomarker for IBD. Genes of interest for colonic inflammation were validated in a second independent experiment using qPCR. Indeed, TNF-α was significantly reduced in mice that received SpNS compared with sham stimulated animals (0.44 vs. 1.00, respectively, *P* = 0.009; Fig. 5B). As we showed in the spleen, genes in the HIF-1α signaling pathway were reduced, although this effect was less pronounced in the colon compared with the spleen (Fig. 5C).

## Discussion

Here, we demonstrate that SpNS induced transcriptional changes in the spleen, and reduced clinical signs of colitis such as colon oedema and histological parameters scored in the assessment of colitis, while having a limited effect on colon inflammation. The results of this study support both *ex* and in vivo studies in models of endotoxemia demonstrating SpNS can be used to reduce inflammatory activation of splenocytes [16, 41, 42]. Recently, the effect of both electrical and ultrasound SpNS was found to improve inflammation in two different animal models of arthritis [9, 43]. Similarly, ultrasound stimulation of the spleen and splenic nerve bundle improved DSS-induced colitis [17]. Although it could not be excluded that other organs were affected by this stimulation, the immune modulatory effects were not observed in splenectomized animals, and therefore, ultrasound was thought to target the spleen and splenic nerve bundle directly. Our results largely corroborate the observations made by Nunes et al. regarding the weight loss and decrease in colon density and histology scoring after stimulation. In line, in our studies a negative regulation of expression of various cytokines by SpNS was noted, depending on the starting time of stimulation after the induction of DSS-induced colitis. This highlights the complexity of the DSS model, in which duration of DSS and moment of outcome measurement vary throughout literature [44, 45]. The fact that both electrical (this study) and ultrasound (Nunes et al.) SpNS reduce disease severity following DSS-induced colitis strengthens the idea that SpNS might hold potential as a therapy for patients with IBD, although further mechanistic studies are warranted.

The parameters used in this study were similar to the stimulation settings that were used in an earlier reported model of experimental arthritis [9]. Interestingly, the anti-inflammatory effects of SpNS in the arthritis experiments were more profound than what we have found in our study. It may well be that the pathology in the collagen-induced arthritis model is primarily dependent on systemic cells and cytokines, and is, therefore, better controlled through SpNS compared to DSS-induced colitis, which is a more mucosal inflammatory process. Noteworthy, Guyot et al. focused on stimulation of a nerve branch that innervated the cranial pole of the spleen, which consists of cholinergic and adrenergic fibers in contrast to the adrenergic, arterial branches. Conversely, in our present study we focused on stimulation of the nerves that run along the artery, because this bundle resembles human anatomy regarding splenic innervation more accurately and is surgically accessible in humans [46, 47].

While VNS has been found to result in anti-inflammatory effects in various experimental conditions, such as sepsis, postoperative ileus, rheumatoid arthritis and kidney disease, we were unable to demonstrate any anti-inflammatory effects of VNS in our DSS-induced colitis model [48–51]. Histologic examination of the cuff showed intact neural tissue and stimulation parameters used were similar to other studies, making it unlikely that VNS was taking place insufficiently, or vagus nerve bundles were damaged [15, 52]. There could be several other explanations for this contradiction. In earlier studies VNS was shown efficacious in reducing colitis in rats under 2,4,6-trinitrobenzene sulfonic acid (TNBS)-induced colitis and only improved survival from oxazolone-induced colitis in mice, both representing more detrimental models of colitis [6, 53]. For the DSS-induced colitis model no beneficial effect of stimulation has been reported previously, although vagotomy did worsen colitis [37]. Furthermore, VNS did not improve the histology score in all studies, which is the conventional outcome parameter for experimental colitis [4]. While we could not replicate the anti-inflammatory effect of VNS, it still holds potential as it seems to improve outcome of patients with Crohn’s disease in a limited, non-randomized controlled clinical trial [2]. The off-target effects such as heart rate depression remain undesirable, but this might be prevented by application of subdiaphragmatic VNS [7].

Although we discuss indirect modulation of colitis through the splenic nerve plexus and vagus nerve, it should be noted that sympathetic nerve stimulation or stimulation of other nerves directly innervating the colon such as the superior mesenteric nerve and sacral nerves have all been shown to improve experimental colitis in earlier studies [54–56]. This underlines the potential of neuromodulation as a treatment for IBD patients.

Our analysis showed that inflammatory gene transcription during DSS-induced colitis is counteracted by SpNS in spleen and colon. Our approach is one of a preventive intervention into DSS-induced colitis, rather than a treatment of an established colitis, which is a limitation of this study. Another drawback of the current study is that the observed signal could well be the result of an interplay between the proportional representation of various cell types as well as changes in their transcriptional profiles. From earlier studies we know that SpNS causes transcriptional changes in the T and B cell populations present in the spleen [43]. However, we previously demonstrated that the AR activity affects the macrophage potential to adapt their metabolic profile in vitro, supporting our interpretations in the current study [38]. Inflammatory macrophage activation blunts oxidative phosphorylation, thereby preventing repolarization, strictly directing inflammatory cell expression [39]. Having identified SpNS-associated differences in the expression of HIF-1α and oxidative phosphorylation, it is enticing for us to suggest that SpNS affects macrophage activation quite specifically. This is also supported by the scRNA-seq results showing a large portion of macrophages expressing *Adrb2*. The latter could at least in part provide a mechanism for the beneficial action of splenic nerve bundle activation on immune driven pathology, such as in colitis or collagen-induced arthritis [9]. Nonetheless, future studies are necessary to disentangle which SpNS-associated differences are the result of cellular heterogeneity and the transcriptome thereof.

## Conclusions

We demonstrated the effects of SpNS in a murine model of colitis and showed that SpNS ameliorated clinical features of colitis and caused differential expression of genes in the spleen and colon involved in inflammation. Future experimental studies should focus on the connection between the spleen and colon to gain more insight in the therapeutic mechanisms of SpNS. Meanwhile, a clinical trial in patients undergoing esophagectomy will investigate safety and feasibility of SpNS for application in humans (www.clinicaltrials.gov; NCT04171011).

## Supplementary Information

**Additional file 1. **SplX did not affect outcome of DSS-induced colitis. [A] Bodyweight loss of mice over time, compared with day 0. Data are expressed as mean and standard deviation (SD). [B] Disease activity index. [C] Colon length. [D] mRNA levels of IL-1β, IL-6 and TNF-α. mRNA levels are normalized against the reference genes *Cyclophilin* and *RPLP0*. *N* = 8–10 per group. Data are expressed as mean or median and individual data points. * indicates a significant difference compared to control. Statistical differences between control and DSS-treated mice and between sham mice and SplX were assessed using an independent t-test or a Mann–Whitney U test. *P* < 0.05 was considered significant. DSS: dextran sulfate sodium; Splx: absence of splenic innervation; TNF: tumor necrosis factor; IL: interleukin; LPS: lipopolysaccharide; RPLP0: Ribosomal Protein Lateral Stalk Subunit P0.

**Additional file 2. **VNS does not affect DSS-induced colitis. [A] Bodyweight loss of mice over time, compared with day 0. Data are expressed as mean and SD. [B] Histology score. [C] Colon weight/length ratio, endoscopy score and DAI. [D] mRNA levels of TNF-α, IL-6, IL-1α, IL-1β, IL-10, IL-12. mRNA levels are normalized for reference genes *Nono* and *RPLP0*. *N* = 16, only female mice. Data are expressed as median and individual data points. Statistical differences between sham and stim mice were assessed using an independent Mann–Whitney *U* test. *P* < 0.05 was considered significant. VNS: vagus nerve stimulation; TNF: tumor necrosis factor; IL: interleukin; Nono: Non-POU domain-containing octamer-binding protein; RPLP0: Ribosomal Protein Lateral Stalk Subunit P0.

**Additional file 3.** Implanted cuff electrodes in this study. [A] Picture of implanted 100 µm micro cuff sling (CorTec GmbH) around splenic nerve bundle. [B] Scheme of electrical pulse parameters used.

**Additional file 4. **Schematic overview of experimental setup for SpNS. Differentially expressed genes were assessed between colonic samples of mice that did not receive stimulation or DSS, sham stimulated mice with DSS-induced colitis, and SpNS-treated mice with DSS-induced colitis. SpNS: splenic nerve bundle stimulation; DSS: dextran sulfate sodium.

**Additional file 5. **Transcriptomic changes in the colons from colitic mice. Volcano plot of the –log_10_(-*P*) on the Y-axis vs. the mean log2 fold change on the X-axis of mice that received DSS vs. control mice. Genes that were significantly different are indicated in blue (downregulated) and red (upregulated). DSS: dextran sulfate sodium; TNF: tumor necrosis factor; IL: interleukin; MMP: matrix metalloproteinase; ACOD: cis-aconitate decarboxylase; CXCL: chemokine (C–X–C motif) ligand; S100: S100 calcium-binding protein.

## Data Availability

All data and materials are available from the corresponding author upon request.

## Abbreviations

β2-AR: β2-Adrenergic receptor
DAI: Disease activity index
DSS: Dextran sulfate sodium
HIF-1α: Hypoxia-Inducible Factor 1 alpha
IBD: Inflammatory bowel diseases
MMP: Matrix metalloproteinase
NE: Norepinephrine
Nos2: Nitric oxide synthase 2
SplX: Disruption of splenic nerve bundle
SpNS: Splenic nerve bundle stimulation
TNF-α: Tumor Necrosis Factor α
VNS: Vagus nerve stimulation
