# Supplementary figures and images for: Electrical stimulation of the splenic nerve bundle ameliorates dextran sulfate sodium-induced colitis in mice
Source: J Neuroinflammation. 2022 Jun 17;19:155. doi: 10.1186/s12974-022-02504-z (PMC9204975; doi:10.1186/s12974-022-02504-z)

**A**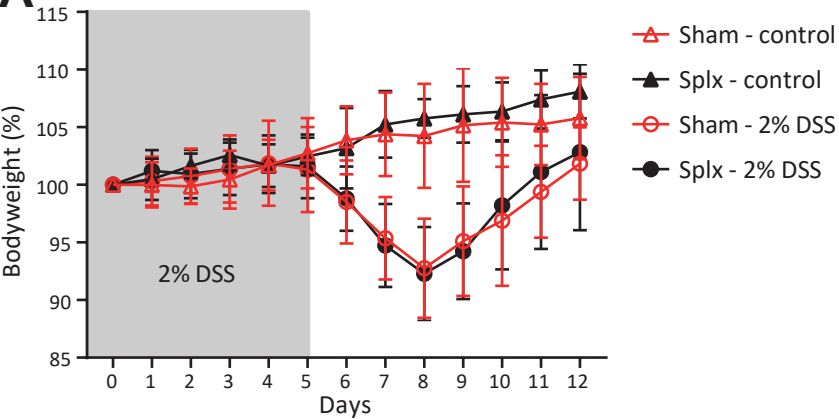**B**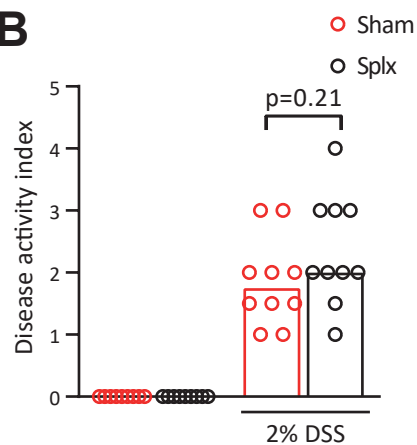**C**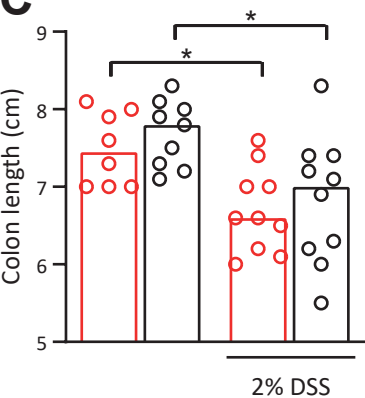**D**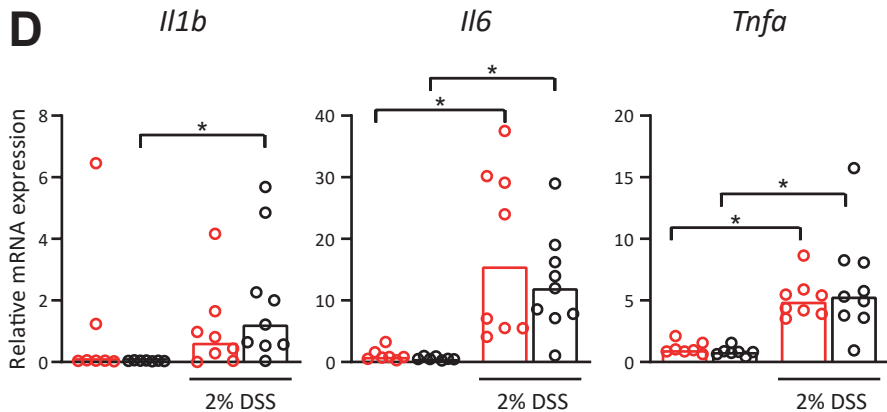

Supplement: Supplementary file 1 — Additional file 1. SplX did not affect outcome of DSS-induced colitis. [A] Bodyweight loss of mice over time, compared with day 0. Data are expressed as mean and standard deviation (SD). [B] Disease activity index. [C] Colon length. [D] mRNA levels of IL-1β, IL-6 and TNF-α. mRNA levels are normalized against the reference genes Cyclophilin and RPLP0. N = 8–10 per group. Data are expressed as mean or median and individual data points. * indicates a significant difference compared to control. Statistical differences between control and DSS-treated mice and between sham mice and SplX were assessed using an independent t-test or a Mann–Whitney U test. P < 0.05 was considered significant. DSS: dextran sulfate sodium; Splx: absence of splenic innervation; TNF: tumor necrosis factor; IL: interleukin; LPS: lipopolysaccharide; RPLP0: Ribosomal Protein Lateral Stalk Subunit P0. [file 12974_2022_2504_MOESM1_ESM.pdf]

**A**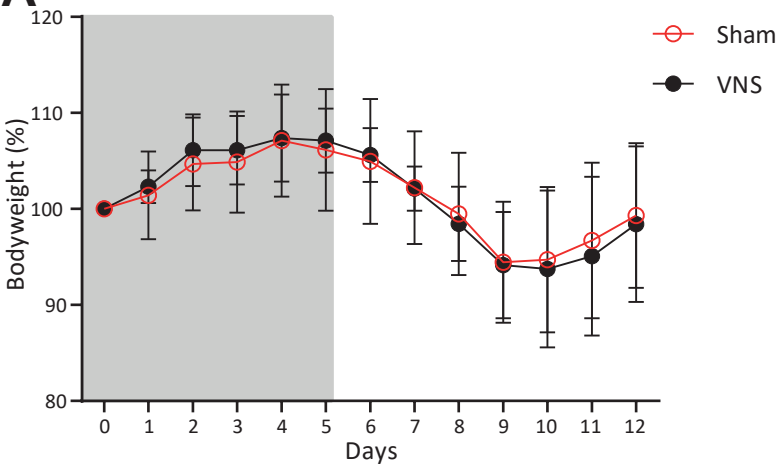**B**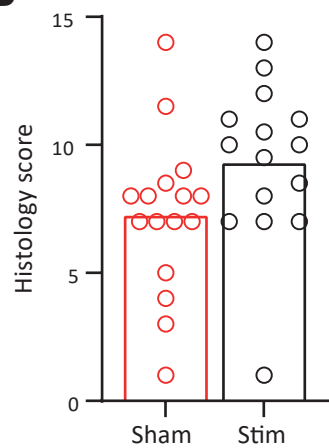**C**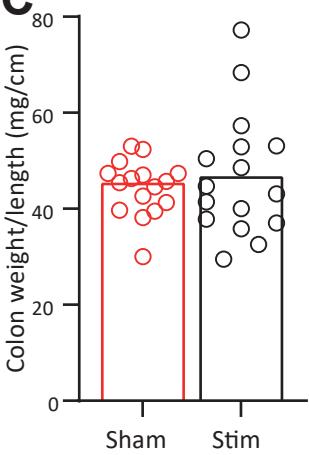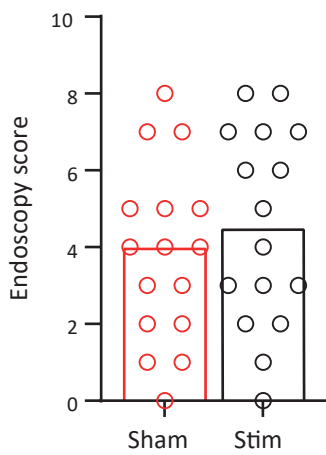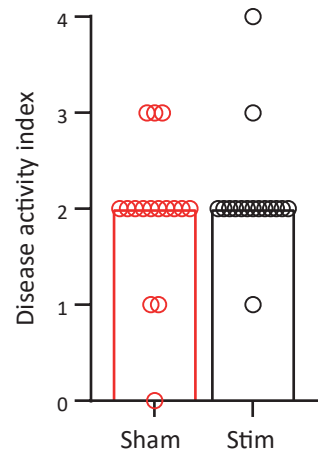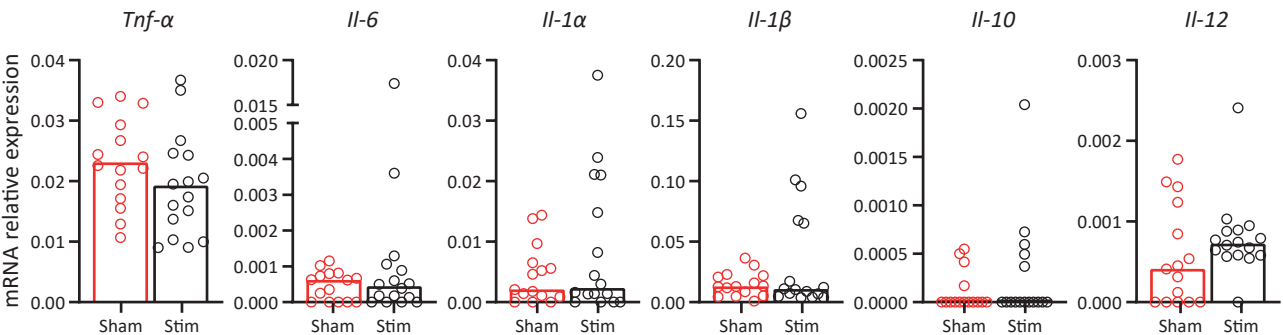

Supplement: Supplementary file 2 — Additional file 2. VNS does not affect DSS-induced colitis. [A] Bodyweight loss of mice over time, compared with day 0. Data are expressed as mean and SD. [B] Histology score. [C] Colon weight/length ratio, endoscopy score and DAI. [D] mRNA levels of TNF-α, IL-6, IL-1α, IL-1β, IL-10, IL-12. mRNA levels are normalized for reference genes Nono and RPLP0. N = 16, only female mice. Data are expressed as median and individual data points. Statistical differences between sham and stim mice were assessed using an independent Mann–Whitney U test. P < 0.05 was considered significant. VNS: vagus nerve stimulation; TNF: tumor necrosis factor; IL: interleukin; Nono: Non-POU domain-containing octamer-binding protein; RPLP0: Ribosomal Protein Lateral Stalk Subunit P0. [file 12974_2022_2504_MOESM2_ESM.pdf]

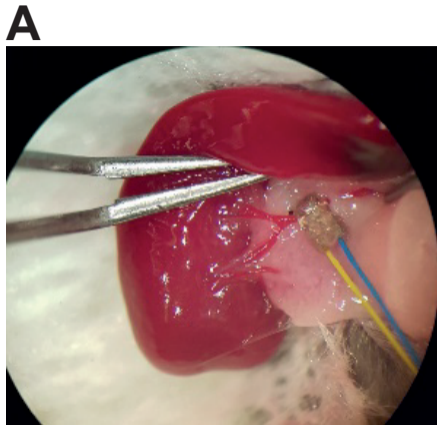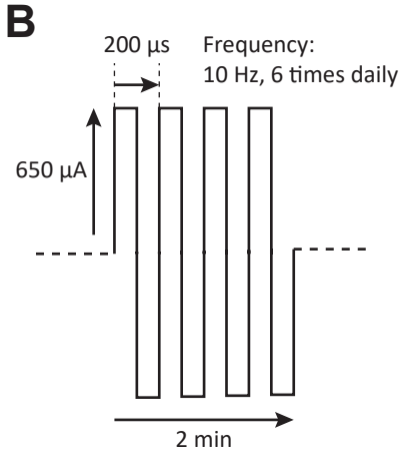

Supplement: Supplementary file 3 — Additional file 3. Implanted cuff electrodes in this study. [A] Picture of implanted 100 µm micro cuff sling (CorTec GmbH) around splenic nerve bundle. [B] Scheme of electrical pulse parameters used. [file 12974_2022_2504_MOESM3_ESM.pdf]

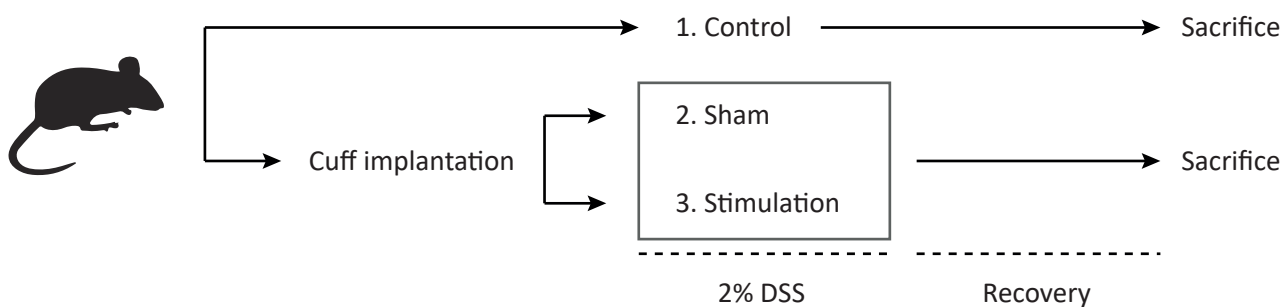

Supplement: Supplementary file 4 — Additional file 4. Schematic overview of experimental setup for SpNS. Differentially expressed genes were assessed between colonic samples of mice that did not receive stimulation or DSS, sham stimulated mice with DSS-induced colitis, and SpNS-treated mice with DSS-induced colitis. SpNS: splenic nerve bundle stimulation; DSS: dextran sulfate sodium. [file 12974_2022_2504_MOESM4_ESM.pdf]

# Colon: DSS-Sham vs Control

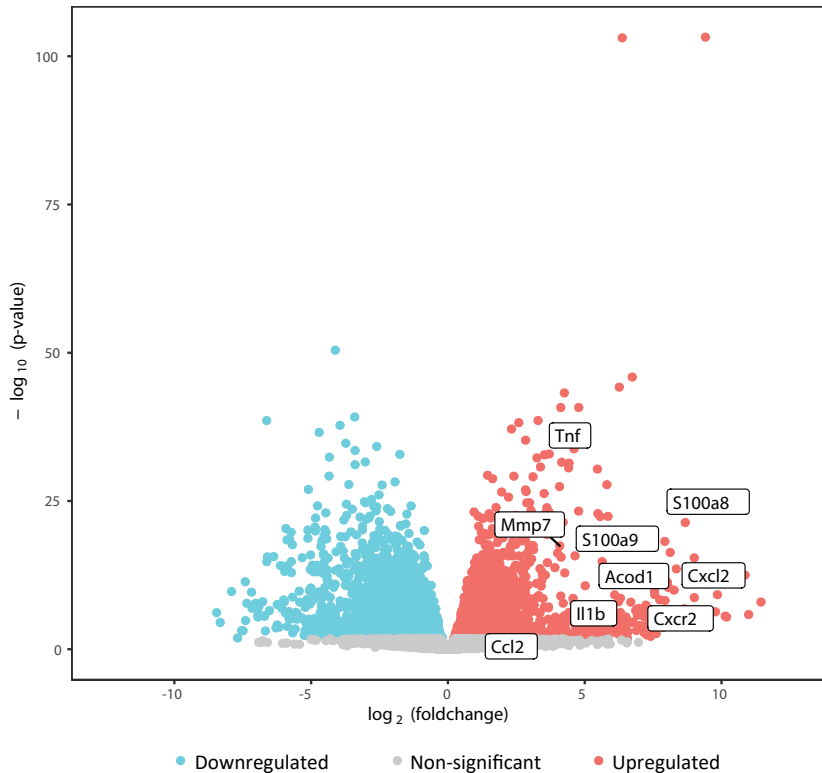

Supplement: Supplementary file 5 — Additional file 5. Transcriptomic changes in the colons from colitic mice. Volcano plot of the –log10(-P) on the Y-axis vs. the mean log2 fold change on the X-axis of mice that received DSS vs. control mice. Genes that were significantly different are indicated in blue (downregulated) and red (upregulated). DSS: dextran sulfate sodium; TNF: tumor necrosis factor; IL: interleukin; MMP: matrix metalloproteinase; ACOD: cis-aconitate decarboxylase; CXCL: chemokine (C–X–C motif) ligand; S100: S100 calcium-binding protein. [file 12974_2022_2504_MOESM5_ESM.pdf]
